# Unearthing the Potential Therapeutic Effects of Oxyresveratrol Based on Intrinsic Links between Pharmacological Effects: Implications for the Gut–Liver–Brain Axis

**DOI:** 10.3390/ph17081063

**Published:** 2024-08-13

**Authors:** Lijuan Zhao, Yan Duan, Zhaoxing Li, Juan Li, Shunxiang Li

**Affiliations:** 1 School of Pharmacy, Hunan University of Chinese Medicine, Changsha 410208, China; 20202094@stu.hnucm.edu.cn (L.Z.); 20222057@stu.hnucm.edu.cn (Y.D.); forever_lijuan@163.com (J.L.); 2 Hunan Engineering Technology Research Center for Bioactive Substance Discovery of Chinese Medicine, Changsha 410208, China; 1520220126@cpu.edu.cn; 3 Hunan Province Sino-US International Joint Research Center for Therapeutic Drugs of Senile Degenerative Diseases, Changsha 410208, China; 4 College of Biology and Food Engineering, Huaihua University, Huaihua 418000, China; 5 School of Pharmacy, China Pharmaceutical University, Nanjing 210009, China

**Keywords:** oxyresveratrol, stilbene compounds, molecular docking, gut–liver–brain axis, synergistic effect

## Abstract

Oxyresveratrol is a stilbene compound with a simple chemical structure and various therapeutic potentials. This study summarized and analyzed the multiple pharmacological effects and mechanisms of oxyresveratrol, identifying its prominent performance in neuroprotection, hepatoprotection, and anti-inflammatory activities in the intestines. By integrating the pharmacological effects of oxyresveratrol with insights from the network pharmacology and molecular docking of its interactions with targets linked to gut–liver–brain axis disorders, it has been shown that oxyresveratrol may hold promise for the treatment of gut–liver–brain axis-related disorders. The synergistic effect between various mechanisms has inspired further research and the development of oxyresveratrol’s application value.

## 1. Introduction

**Figure 1:**
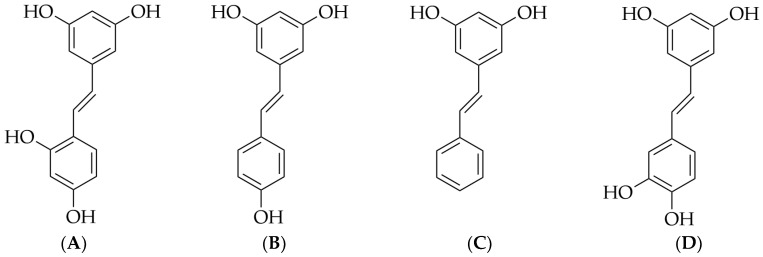
Chemical structures of oxyresveratrol (**A**), resveratrol (**B**), pinosylvin (**C**), and piceatannol (**D**).

Oxyresveratrol (4-[(E)-2-(3,5-dihydroxyphenyl)ethenyl]benzene-1,3-diol), a small-molecule stilbene compound, is known for its diverse pharmacological activities. Its chemical structure is depicted in Figure 1A. Oxyresveratrol is naturally present in gymnosperm plants of the Gynaceae family and has been identified in various species of angiosperms, including 6 species of plants, 9 species of monocots, and 38 species of dicots [1]. Plants known to contain oxyresveratrol include Artocarpus lakoocha Roxb., *Smilax poria*, *Veratrum nigrum* L., *Gnetum montanum* Markgr., Mori Cortex, and Ramulus Mori [2,3]. Stilbene compounds are a type of plant secondary metabolite, and their synthetic pathway relies on the key enzyme stilbene synthase. Some scholars believe that resveratrol (Figure 1B) and similar compounds, such as pinosylvin (Figure 1C) and piceatannol (Figure 1D), are precursors for all stilbene derivatives, including oxyresveratrol. However, more specific experimental evidence is needed to support this claim [1].

Oxyresveratrol and resveratrol have similar structures. Oxyresveratrol exhibits a higher vital clearance ability and tissue permeability than resveratrol [4,5]. According to the latest research, oxyresveratrol expresses tyrosinase inhibitory activity, suppresses melanin production, and exhibits a whitening function. It also possesses antibacterial, anti-inflammatory, antioxidant, anti-apoptotic, neuroprotective, anti-hyperglycemic, and cancer prevention and treatment properties [6,7,8,9]. This review summarizes oxyresveratrol’s reported pharmacological activities and mechanisms of action and reveals its outstanding performance in protecting the nervous system, combating liver diseases, and reducing intestinal inflammation. By considering the integration of the pathways of oxyresveratrol’s targets, this review suggests that oxyresveratrol might potentially have effects on the gut–liver–brain axis. This study aimed to provide new ideas and insights for the overall in-depth study of oxyresveratrol’s pharmacological effects.

## 2. Oxyresveratrol’s Pharmacological Effects and Mechanisms

### 2.1. Oxyresveratrol’s Anti-Cancer and Anti-Malignant Tumor Effects

Cancer occurrence is a highly complex process. The influences of proto-oncogene activation, cell proliferation regulation, and the disorder of normal apoptosis programs might cause carcinogenesis [10]. Evidence suggests that oxyresveratrol displays anti-cancer effects via multiple pathways. Firstly, oxyresveratrol has a direct toxic effect on ovarian cancer, lung cancer, and cervical cancer cell lines by binding to the DNA of cancer cells, altering the DNA structure, and causing cancer cell death [11,12,13]. Secondly, oxyresveratrol inhibits cancer cell growth and proliferation by affecting the replication and repair of DNA in the G0/G1 phase and S phase of the cell cycle, significantly downregulating the expression of the DNA repair protein RAD51 gene (Rad51), which is related to the DNA homologous recombination repair pathway [14,15]. Thirdly, oxyresveratrol induces apoptosis in a variety of cancer cells. Studies showed that oxyresveratrol application led to the apoptosis of neuroblastoma cells (SH-SY5Y) and breast cancer cells (MDA-MB-231) [12,16]. Additionally, research has indicated that oxyresveratrol inhibits cell viability and induces apoptosis in osteosarcoma (Saos-2) cells [17].

Culturing with oxyresveratrol significantly inhibited cancer cell migration in colorectal cancer cells (CRCs) [18,19] and also in the liver cancer cell lines QGY-7701 and SMMC-7721. Oxyresveratrol also inhibited the tumor growth of hepatocellular carcinoma induced by H22 cells in a dose-dependent manner [20].

Oxyresveratrol could achieve its effects by increasing the number of normal cells, reducing apoptosis, and scavenging free radicals, especially in mitochondrial protection [21,22,23,24,25,26]. Mitochondria are the primary targets of oxidative damage and the apoptotic pathways [27], and mitochondrial dysfunction is a pathological manifestation of various diseases [28,29,30]. Trans-crotonaldehyde (TCA) is the molecule responsible for mitochondrial lipid metabolism and is an essential toxic product of oxidation [31,32]. Toxic TCA is considered to attack mitochondrial DNA [33,34] and is closely related to the molecular mechanism of cancer formation [35]. Studies have found that oxyresveratrol scavenges the aldehyde group (—CH=O) of mitochondrial toxic TCA to protect mitochondria [36].

In summary, oxyresveratrol exerts anti-cancer effects by directly damaging the DNA of cancer cells, and by inhibiting cancer cell proliferation and metastasis [37]. In addition, oxyresveratrol could ameliorate immunity by enhancing the cellular vitality of normal cells.

### 2.2. Oxyresveratrol’s Inhibiting Effect on Melanin Formation

Excessive melanin deposition can lead to esthetic skin problems [38]. Elevated levels of reactive oxygen species (ROS) activate the ɑ-melanocyte-stimulating hormone in the epidermis to activate tyrosinase (TYR). This is regulated by melanogenesis-associated transcription factor (MITF), which catalyzes the hydroxylation of tyrosine to 3,4-dihydroxyphenylalanine (DOPA) [4]. DOPA is then oxidized to dopaquinone, and its derivatives are oxidatively polymerized to produce melanin [39]. Recent studies have demonstrated that oxyresveratrol inhibits the gene transcription and protein expression of the TYR gene family by suppressing the expressions of MITF and TYR-related protein-2 (TRP-2), thereby reducing the additive effect of melanin [4]. Additionally, oxyresveratrol functions as a non-competitive inhibitor of TYR, leading to decreased TYR activity and melanin content by facilitating ROS removal in cells [40,41]. Oxyresveratrol has multi-target and multi-link inhibitory effects on the production process of melanin and age pigments [42,43,44].

### 2.3. Oxyresveratrol’s Protective Effect on the Nervous System

With the intensification of global aging, the treatment of neurodegenerative diseases, especially Parkinson’s disease (PD) and Alzheimer’s disease (AD), has attracted increasing attention [45,46]. Endoplasmic reticulum stress (ERS) is a critical mechanism of PD pathology and triggers the pathways, showing protective effects in the early stages [47,48]. However, when the damage expands, apoptosis is triggered [49]. Various PD models have shown that oxyresveratrol significantly reduces the release of lactate dehydrogenase and the activity of cysteine-containing aspartate-specific protease-3 (caspase-3) by reducing ERS and inhibiting the transcription of activated transcription factor-4 (ATF4) via other pathways to reduce nerve cell apoptosis [50,51]. The amyloid precursor protein (APP) is a hallmark of AD, as the precursor of β-amyloid. In mouse cortical astrocytes, oxyresveratrol reduced APP by regulating AMP-activated protein kinase (AMPK)/unc-51-like autophagy activating the kinase-1 (ULK1)/mammalian target of rapamycin (mTOR)-dependent induction of autophagy, and significantly decreased neuronal cell loss [52,53]. In addition, oxyresveratrol works to protect cortical and hippocampal neurons from damage by β-amyloid.

Neuroinflammation is closely related to the occurrence and development of PD and AD [54,55]. Oxyresveratrol has anti-neuroinflammation effects, and it significantly reduces the release of IL-6 and MCP-1 in HMC3 cells stimulated by IL-1β and inhibits the activation of the PI3K/AKT/p70S6K pathway induced by IL-1β [56]. Moreover, oxyresveratrol effectively suppresses the release of pro-inflammatory mediators from BV-2 cells stimulated by lipopolysaccharide (LPS) and then exerts anti-inflammatory effects via the MAPKs and NF-*κ*B signaling pathways [57,58]. Furthermore, it has demonstrated protective effects on various neural cell injury models, including acute hippocampal neuron cell death induced by kainic acid (KA), ethanol-induced DNA damage in the mouse cerebellum and cerebral cortex, and H_2_O_2_-induced PC12 cell-death experiments [59,60].

### 2.4. Oxyresveratrol’s Anti-Obesity Effect

Obesity is a hidden trouble that can lead to various diseases [61]. Thermogenesis is a new method to fight obesity, wherein the energy is consumed as calories instead of being stored as lipids [62]. The acceleration of mitochondrial biogenesis and the expression of thermogenesis-related genes in subcutaneous white adipose tissue initiates a browning program, resulting in the formation of beige adipose tissue [63]. Subsequently, beige fat converts energy into heat dissipation, presenting a novel strategy for preventing and treating obesity by inducing the beige coloration of white adipose tissue and enhancing energy expenditure [64]. In experiments, oxyresveratrol sped up energy conversion by increasing the expression of the thermogenesis-related uncoupling protein (UCP1) in adipose tissue and significantly activated carnitine palmitoyl transferase-1 (CPT1) [65]. In addition, oxyresveratrol accelerated the beige coloration of white adipose tissue by decreasing lipid accumulation and the expression of adipocyte markers during the differentiation of 3T3-L1 and C3H10T1/2 adipocytes, inducing thermogenic genes and inhibiting white adipocyte selection genes [66,67]. Oxyresveratrol treatment in obese mice that were fed a high-fat diet significantly reduced adipose tissue weight, prevented weight gain, and alleviated obesity-related symptoms.

### 2.5. Oxyresveratrol’s Protective Effect on the Liver

Liver damage can cause serious harm to the body, ranging from fatty liver to liver fibrosis and cirrhosis, which can eventually lead to liver failure or liver cancer [68,69]. Oxyresveratrol significantly reduces serum alanine aminotransferase (ALT) and aspartate aminotransferase (AST) levels in different liver injury models [70]. It also decreases the expression of inflammatory factors and inhibits the liver toll-like receptor 4 (TLR4)/NF-*κ*B signaling pathway, helping to prevent liver cell degeneration and inflammatory cell infiltration [71]. It also inhibits the expression and activation of caspases, reduces the hepatocyte apoptosis stimulated by galactosamine (LPS/d-GalN), and blocks the generation of ROS and the cell death of hepatocytes induced by tert-butyl hydroperoxide (TBHP) [72].

Non-alcoholic fatty liver disease (NAFLD) is one of the world’s most prevalent liver diseases. Currently, few drugs can be used clinically to treat NAFLD [73]. In hepatocyte models, it was observed that oxyresveratrol could inhibit the induction of sterol regulatory element-binding proteins (SREBP-1C) by liver X receptor (LXR) agonists. This led to a downregulation of lipid genes, while the genes related to fatty acid oxidation were promoted in hepatocytes. Additionally, liver lipogenesis was reduced, and fatty liver disease formation was prevented [74].

Oxyresveratrol not only relieves the symptoms of liver damage and non-alcoholic fatty liver disease but also affects the process of hepatic fibrosis (HF). The activation of hepatic stellate cells (HSCs) is the most critical event in HF. Yes-associated protein 1 (YAP) and transforming growth factor β1 (TGF-β1) may be critical regulators of hepatic stellate cell (HSC) activation. Oxyresveratrol acts on the Hippo/YAP and TGF-β1/Smad signaling pathways to influence liver fibrosis [75].

### 2.6. Oxyresveratrol’s Protective Effect on the Intestines

The intestinal tight junction (TJ) ensures the integrity of the intestinal mucosa, provides an effective barrier for the normal absorption of nutrients, and protects against intestinal pathogens, allergens, and toxins [76,77]. It is also vital in the recovery from inflammatory bowel disease (IBD) [78]. Oxyresveratrol enhances the expression of intestinal tight junction proteins and the integrity of the intestinal TJ barrier [79,80]. Furthermore, oxyresveratrol stimulates the expression of mucoprotein 2 (MUC2) in human intestinal goblet cells, maintaining and renewing the intestinal mucus to ensure the stability of the intestinal mucus barrier [81,82,83].

### 2.7. Oxyresveratrol’s Antibacterial Effect

Oxyresveratrol has an inhibitory effect on bacteria and fungi. The minimum inhibitory concentration (MIC) of oxyresveratrol to *Staphylococcus aureus* is 128–256 μg/mL [84]. In addition, oxyresveratrol has a dose-dependent inhibitory effect on various oral bacteria, such as *Streptococcus mutans* and *Streptococcus gordonii*, and could exert antibacterial effects by significantly downregulating glucosyltransferase expression, inhibiting glucan synthesis, affecting biofilm formation, and eventually reducing the survival rate of *Streptococcus mutans* [85,86,87]. Methicillin-resistant *Staphylococcus aureus* (MRSA) treatment with oxyresveratrol was found to promote cell membrane permeability and inhibit growth and reproduction [88,89]. Oxyresveratrol can suppress the bacterial production, population movement, and agglutination ability of Gram-negative bacteria, the uropathogenic *Escherichia coli* (UPEC), by inhibiting UPEC biofilm formation [90]. In addition, oxyresveratrol initiates the mitochondria-related apoptotic pathway by activating the mitochondria-mediated apoptosis of *Candida albicans*, and it has an antifungal effect by inhibiting the activity of *Trichophyton rubrum* [91,92].

### 2.8. Oxyresveratrol’s Anti-Inflammatory Effect

Inflammation is a primary pathological reaction that can be related to many diseases [93,94]. The persistent presence and high expression of inflammatory mediators can trigger cascade reactions, such as inducing cell proliferation and increasing ROS production [95]. Oxyresveratrol effectively suppresses the inflammatory response triggered by LPS in an estrogen receptor (ER)-dependent manner by modulating the NF-*κ*B signaling pathway [96,97,98,99]. This modulation leads to a decreased expression of inflammatory factors and a reduced production of matrix metalloproteinase 13 (MMP-13), thus attenuating the inflammatory response [100]. In an alcoholic ulcer mouse model, oxyresveratrol had a significant inhibitory effect on inflammatory infiltration and ulcers and exerted an anti-inflammatory effect by markedly reducing the transcription levels of various pro-inflammatory factors [101]. In a skin inflammation model, oxyresveratrol reduced the number of CD3, CD4, and CD8 T cells in the sensitized skin of mice [102]. Oxyresveratrol could improve the inflammatory status of dermatitis models, both in vitro and in vivo. It effectively inhibited excessive cell proliferation by downregulating TNF-α in a dose-dependent manner in the keratinocytes and also inhibited AKT phosphorylation [103].

### 2.9. Oxyresveratrol’s Effect on Blood Sugar Regulation

Blood sugar is an essential indicator of physical health as related to insulin and glucagon secretion [104,105,106]. Oxyresveratrol enhances insulin secretion in INS-1 cells and has shown an anti-glycosylation effect by capturing methylglyoxal and inhibiting the production of advanced glycation end products (AGEs) [107,108,109]. It might also regulate blood sugar by improving β-cell dysfunction and insulin resistance and stabilizing or enhancing the activity and expression of glucokinase (GK) [66,110]. Blood sugar levels were significantly reduced in diabetic ICR mice treated with oxyresveratrol because it inhibited maltose hydrolysis and reduced intestinal cell glucose transport [111,112,113].

### 2.10. Oxyresveratrol’s Other Pharmacological Effects

Oxyresveratrol’s pharmacological effects indicate its ability to regulate and improve various bodily functions. In addition, oxyresveratrol undergoes metabolic transformations via double-bond reduction, dihydroxylation, and demethylation under the mediation of colonic microbiota, affecting the species and quantity of intestinal endophytic bacteria [114,115]. The oxidative stress induced by hydrogen peroxide (H_2_O_2_) causes apoptosis in human lens epithelial cells (HLECs) and triggers cataract formation. Oxyresveratrol has a specific protective effect on cataracts by reversing the oxidative stress and apoptosis of HLECs induced by H_2_O_2_ [116].

## 3. Oxyresveratrol and the Gut–Liver–Brain Axis

### 3.1. Gut–Liver–Brain Axis

Based on the summary of oxyresveratrol’s pharmacological effects and mechanisms, oxyresveratrol shows outstanding performance in protecting the nervous system, treating liver injury and intestinal inflammation, and affecting the activity of intestinal microorganisms. According to this evidence, oxyresveratrol may potentially act on the gut–liver–brain axis.

The gut–liver–brain axis is a complex network within the body, involving dialog between various systems such as the gastrointestinal tract, liver, and central nervous system [117,118]. It is implicated in multiple diseases and significantly affects human health. Experimental findings have shown intestinal material leakage occurring in various liver diseases, with significantly elevated levels of endotoxins and lysophosphatidic acid in the patients’ circulatory systems [119,120,121,122,123,124,125,126]. This might activate the neuroinflammatory processes, leading to neurological complications [127,128,129]. Additionally, the interaction between the gut and brain via the nervous and circulatory systems has been observed in various disorders related to the gut–brain axis, indicating that the dysbiosis of the gut microbiota and accumulation of toxic substances from the intestines are closely associated with disease progression [130,131,132,133,134]. Meanwhile, the neurons and the brain provide feedback information to the liver via the vagus nerve’s parasympathetic branch, which also innervates the intestines. Thus, intestinal tract–liver–central nervous system interactions influence various diseases. The inherent beneficial interactions within the gut–liver–brain axis contribute to the nervous system’s development and maintenance [135].

The main drug treatments for diseases related to the gut–liver–brain axis, such as antibiotics, prebiotics, targeted drugs, and so on, only focus on specific areas at once [136,137,138]. Based on oxyresveratrol’s pharmacological effects, it has been found that oxyresveratrol exhibits significant efficacy in simultaneously treating intestinal dysbiosis, intestinal inflammation, and liver diseases and in providing neuroprotection. This offers hope for a holistic approach to treating diseases associated with the gut–liver–brain axis. Network pharmacology and molecular docking studies have been conducted to determine the relationship between oxyresveratrol and the gut–liver–brain axis regarding the related targets and pathways.

### 3.2. Network Pharmacological Research

Eighty-seven targets of oxyresveratrol were found after screening and deduplication via the Traditional Chinese Medicine Systems Pharmacology Database Analysis Platform (TCMSP) and the SwissTargetPrediction website. In total, 4566 targets relating to the gut–liver–brain axis were obtained after screening and deduplication via the GeneCard Database and DisGeNET website.

**Figure 2:**
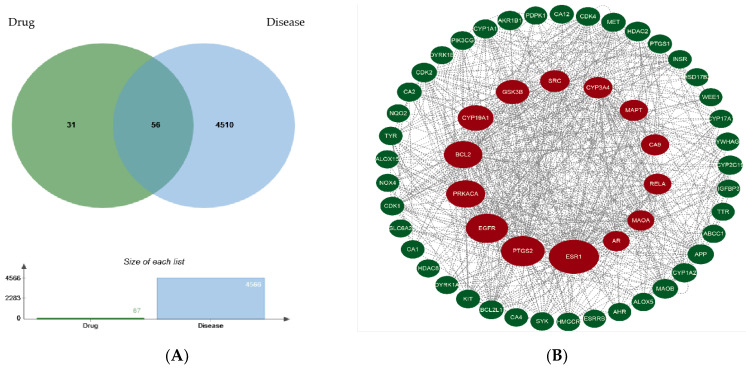
Screening of the common targets of oxyresveratrol (drug) and the gut–liver–brain axis (disease) (**A**) and PPI network construction for the screened targets (**B**).

Fifty-six target genes were obtained when oxyresveratrol’s targets and the gut–liver–brain axis-related targets intersected on the jvenn platform (https://jvenn.toulouse.inrae.fr/app/example.html, accessed on 13 January 2024), as shown in Figure 2A. The overlapping target library was imported into the String website, then Cytoscape 3.9.1 software was used to visually analyze the protein–protein interaction (PPI) network to obtain Figure 2B (the circle’s size in the figure represents the betweenness centrality from large to small).

After calculation using the CytoNCA plug-in of the Cytoscape 3.9.1 software, the node scores from the PPI network were screened by the higher-than-median value of betweenness centrality, closeness centrality, degree centrality, eigenvector centrality, local average connectivity-based method centrality, and network centrality to obtain the network core targets ESR1, BCL2, EGFR, PTGS2, GSK3B, AR, and SRC, as shown in Table 1.

**Figure 3:**
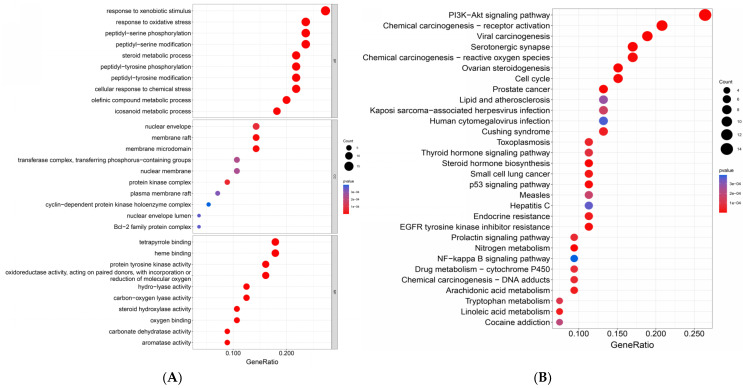
GO enrichment analysis for key targets (**A**) and KEGG pathway enrichment analysis for key targets (**B**).

The top 10 terms in the Biological Process (BC), Cellular Component (CC), and Molecular Function (MF) categories in the Gene Ontology (GO) enrichment analysis of the target genes (Figure 3A) and the top 30 pathways in the *Kyoto Encyclopedia of Genes and Genomes* (KEGG) enrichment analysis (Figure 3B) were obtained using the Rstudio 4.2.3 software. According to the GO enrichment results, oxyresveratrol might affect the gut–liver–brain axis by regulating processes such as peptidyl-serine phosphorylation and modification, the oxidative stress response, and arachidonic acid metabolism in the Biological Process category. In the Cellular Component category, it could influence the cellular membranes and nuclear membrane structures. In the Molecular Function category, oxyresveratrol’s mechanism of action includes protein tyrosine kinase activity; oxidoreductase activity, involving the incorporation or reduction of molecular oxygen; and oxygen binding. These results indicate that oxyresveratrol mainly affects the gut–liver–brain axis by modulating kinase activity and redox reactions.

In the top-30 KEGG pathway analysis shown in Figure 3B, the first enrichment pathway is the PI3K-Akt signaling pathway including 14 out of 54 genes. This pathway and the subsequent P53 pathway are closely related to cell proliferation and cancer development [139,140]. The NF-*κ*B signaling pathway and arachidonic acid metabolism are related to inflammation [141,142]. The serotonergic synapse pathways are related to the nervous system. Additionally, oxyresveratrol’s target genes were also enriched in other pathways related to the gut–liver–brain axis, such as various liver disease pathways, the neurotrophin signaling pathway, the Parkinson’s disease pathway, and cellular adhesion signaling pathways. These enrichment results closely align with oxyresveratrol’s pharmacological effects, as outlined in the preceding section.

### 3.3. Molecular Docking of Oxyresveratrol and Core Targets

**Figure 4:**
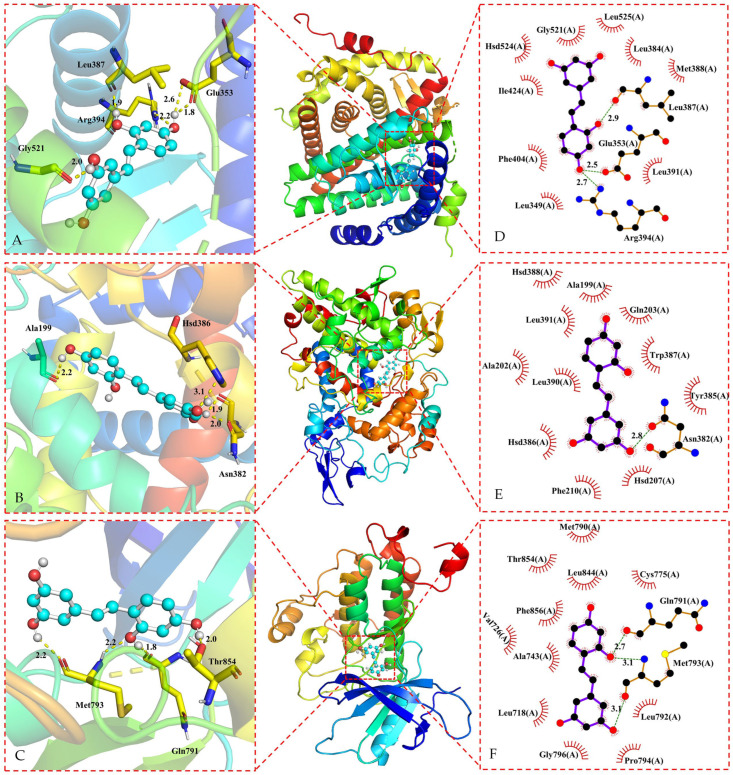
Three-dimensional representations of oxyresveratrol in complex with (**A**) ESR1; (**B**) PTGS2, and (**C**) EGFR. The proteins are shown as colored cartoons, while the oxyresveratrol is represented as cyan sticks. Hydrogen bonds are represented as yellow dashed lines. Two-dimensional representations of oxyresveratrol in complex with (**D**) ESR1; (**E**) PTGS2; and (**F**) EGFR. Hydrogen bonds and hydrophobic contacts are shown as green and red dashed lines, respectively. The ligands are represented as violet lines.

**Figure 5:**
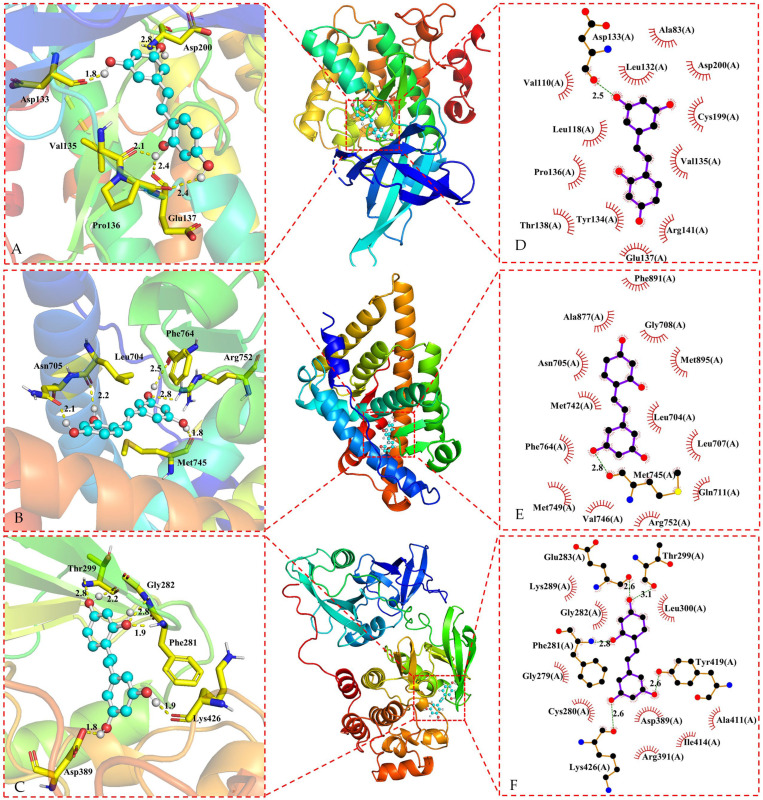
Three-dimensional representations of oxyresveratrol in complex with (**A**) GSK3B; (**B**) AR and (**C**) SRC. The proteins are shown as colored cartoons, while the oxyresveratrol is shown as cyan sticks. Hydrogen bonds are represented as yellow dashed lines. Two-dimensional representations of oxyresveratrol in complex with (**D**) GSK3B; (**E**) AR; and (**F**) SRC. Hydrogen bonds and hydrophobic contacts are shown as green and red dashed lines, respectively. The ligands are represented as violet lines.

Based on the findings presented in Table 1, oxyresveratrol and its core targets were subjected to docking analysis using the LeDock V1.0 software. The binding energy values indicate a strong affinity between oxyresveratrol and most of the core targets, with binding energies below −5.0 kcal/mol [143], except the target BCL2, with the binding energy between BCL2 and oxyresveratrol above −5.0 kcal/mol. The results of the docking process were visualized using the PyMOL 2.5.4 software and the Ligplot+ v.2.2 software, as depicted in Figure 4 and Figure 5.

Oxyresveratrol was able to establish hydrogen bonds and hydrophobic interactions with amino acid residues in ESR1, EGFR, PTGS2, GSK3B, AR, and SRC proteins during the docking process, as evidenced by the data in Figure 4 and Figure 5, and Table 2. Core targets in the KEGG analysis, such as EGFR and GSK3B, were implicated in the PI3K-Akt signaling pathway, hepatocellular carcinoma pathway, and hepatitis C pathway. Meanwhile, SRC was associated with the Gap junction pathway, and PTGS2, EGFR, and GSK3B were identified as key targets in the NF-*κ*B signaling pathway. Additionally, PTGS2 and GSK3B were linked to pathways related to neurodegeneration. There were lower binding energies (<−5.0 kcal/mol) when these targets were docking with oxyresveratrol, indicating a favorable binding affinity between oxyresveratrol and these core targets. The visual representations suggest hydrogen bonds and hydrophobic interactions between oxyresveratrol and amino acid residues of the target proteins during binding, potentially leading to alterations in the proteins’ spatial structures and functional domains, consequently affecting their activity.

## 4. Discussion

Oxyresveratrol achieves a variety of pharmacological activities by acting mainly on inflammation, tight junctions, and cancer pathways [144], the PI3K/AKT signaling pathway [145], insulin regulatory pathways, AD and other neurodegenerative disease pathways, lifespan regulatory pathways, etc. Oxyresveratrol’s effects on anti-intestinal inflammation, liver-injury treatment, and nervous system protection are closely related to the gut–liver–brain axis [146,147,148,149,150].

The study of the gut microbiome–liver–brain axis system model has become the focus of nervous system research [151,152,153], and increasing evidence shows a connection between inflammatory bowel disease, neurodegenerative diseases, and neuroinflammatory diseases [154]. Epidemiological, clinical pharmacological, and nutritional studies have confirmed that oxyresveratrol has various pharmacological effects, such as anti-cancer effects [144,155], protection against oxidative stress and neurodegenerative diseases [156,157], and the treatment of liver, intestinal tract, and nervous system diseases. Dysbiosis of the intestinal microbiota activates the intestinal immune system, thereby enhancing intestinal permeability and bacterial translocation, leading to neuroinflammation, cerebrovascular changes, and the formation of AD-related β-amyloid and PD-related α-synuclein aggregation. In turn, the nervous system can regulate the function of the gastrointestinal tract via the parasympathetic nerves. Oxyresveratrol’s pharmacological effects, which were observed in the experimental studies, along with the network pharmacological research and molecular docking results above, highlight the specific targets and pathways associated with oxyresveratrol. These findings suggest that oxyresveratrol may regulate the pathways related to the gut–liver–brain axis through action-related targets. Although oxyresveratrol has low solubility in water and low stability, it is relatively safe when taken orally [158]. Fortunately, significant progress has been made in the research and improvement of oxyresveratrol pharmacokinetics [159,160,161,162,163]. Therefore, oxyresveratrol has excellent research value and development potential in treating intestinal axis pattern-related diseases.

## 5. Conclusions

From the above summary of the various pharmacological effects of oxyresveratrol, it can be inferred that oxyresveratrol exhibits therapeutic effects for diseases associated with the gut–liver–brain axis. Representations of oxyresveratrol’s pharmacological effects, network pharmacology analysis, and an examination of the molecular docking results of oxyresveratrol and the core targets in the pathways connecting to gut–liver–brain axis-related diseases reveal the multi-directional and multi-target treatment potential of oxyresveratrol for such diseases. However, further comprehensive and in-depth research is required to fully develop oxyresveratrol into a clinically effective drug in the long term.

## Figures and Tables

**Table 1:** Screening of core genes.

Rank	Target Name	Betweenness Centrality	Closeness Centrality	Degree Centrality	Eigenvector Centrality	Local Average Connectivity-Based Method Centrality	Network Centrality
1	ESR1	26.02	1	19	0.31	10.74	19
2	BCL2	21.10	0.95	18	0.30	10.56	17.21
3	PTGS2	17.39	0.90	17	0.28	10.35	15.97
4	EGFR	17.83	0.90	17	0.28	10.12	15.64
5	GSK3B	12.34	0.86	16	0.27	10.38	14.94
6	AR	8.32	0.83	15	0.27	10.40	13.73
7	SRC	5.04	0.76	13	0.24	9.23	11.06

**Table 2:** Molecular docking results of oxyresveratrol and the core targets.

Target	RSCB-PDB Code	Resolution	Binding Energy (/kcal·mol^−1^)	Hydrogen Bond Residues	Hydrophobic Residues
ESR1	8DU8	1.47 Å	−5.88	Glu353, Leu387, Arg394, and Gly521	Leu349, Leu384, Met388, Leu391, Phe404, Ile424, Hsd524 and Leu525
PTGS2	5IKR	2.34 Å	−6.42	Ala199, Asn382 and Hsd386	Ala202, Gln203, Hsd207, Phe210, Tyr385, Trp387, Hsd388, Leu390, and Leu391
EGFR	5HG8	1.42 Å	−5.83	Gln791, Met793, and Thr854	Leu718, Val726, Ala743, Cys775, Met790, Pro794, Gly796, Leu844, and Phe856
GSK3B	7SXJ	1.85 Å	−5.14	Asp133, val135, Pro136, Glu137, and Asp200	Ala83, Val110, Leu118, Leu132, Tyr134, Thr138 Arg141, and Cys199
AR	8E1A	1.20 Å	−6.46	Leu704, Asn705, Met745, Arg752, and Phe764	Leu707, Gly708, Gln711, Met742, Val746, Met749, Ala877, Phe891, and Met895
SRC	8JN8	1.90 Å	−5.92	Phe281, Gly282, Thr299, Asp389, and Lys426	Gly279, Cys280, Glu283, Lys289, Leu300, Arg391, Ala411, and Ile414

## References

[1] Likhitwitayawuid K. (2021). Oxyresveratrol: Sources, Productions, Biological Activities, Pharmacokinetics, and Delivery Systems. Molecules.

[2] Kitisripanya T., Inyai C., Krittanai S., Likhitwitayawuid K., Sritularak B., Ploypradith P., Tanaka H., Morimoto S., Putalun W. (2017). A monoclonal antibody-based immunoassay for the determination of oxyresveratrol from *Artocarpus lacucha* Buch.-Ham. J. Nat. Med..

[3] Liang C., Lim J., Kim S. (2012). Dioscin: A synergistic tyrosinase inhibitor from the roots of Smilax china. Food Chem..

[4] Zeng H.J., Li Q.Y., Ma J., Yang R., Qu L.B. (2021). A comparative study on the effects of resveratrol and oxyresveratrol against tyrosinase activity and their inhibitory mechanism. Spectrochim. Acta Part A Mol. Biomol. Spectrosc..

[5] Qiu F., Komatsu K., Saito K., Kawasaki K., Yao X., Kano Y. (1996). Pharmacological properties of traditional medicines. XXII. Phar-macokinetic study of mulberroside a and its metabolites in rat. Biol. Pharm. Bull..

[6] Akinwumi B.C., Bordun K.M., Anderson H.D. (2018). Biological Activities of Stilbenoids. Int. J. Mol. Sci..

[7] Pereira A.C., Arruda M.S.P., Lemos V.S., Cortes S.F., Da Silva E.A.S. (2012). Inhibition of α-glucosidase and hypoglycemic effect of stilbenes from the Amazonian plant *Deguelia rufescens var urucu* (Ducke) A.M.G. Azevedo (Leguminosae). Planta Med..

[8] Lin T.K., Chen S.D., Chuang Y.C., Lin H.Y., Huang C.R., Chuang J.H., Wang P.W., Huang S.T., Tiao M.M., Chen J.B. (2014). Resveratrol partially prevents rotenone-induced neurotoxicity in dopaminergic SH-SY5Y cells through induction of heme oxygenase-1 dependent autophagy. Int. J. Mol. Sci..

[9] Su P.-S., Doerksen R.J., Chen S.-H., Sung W.-C., Juan C.-C., Rawendra R.D., Chen C.-R., Li J.-W., Aisha, Huang T.-C. (2015). Screening and profiling stilbene-type natural products with angiotensinconverting enzyme inhibitory activity from *Ampelopsis brevipedunculata* var. *hancei* (Planch.) Rehder. J. Pharm. Biomed..

[10] Jassim A., Rahrmann E.P., Simons B.D., Gilbertson R.J. (2023). Cancers make their own luck: Theories of cancer origins. Nat. Rev. Cancer.

[11] Chatsumpun N., Chuanasa T., Sritularak B., Lipipun V., Jongbunprasert V., Ruchirawat S., Ploypradith P., Likhitwitayawuid K. (2016). Oxyresveratrol: Structural Modification and Evaluation of Biological Activities. Molecules.

[12] Songoen W., Phanchai W., Brecker L., Wenisch D., Jakupec M.A., Pluempanupat W., Schinnerl J. (2021). Highly Aromatic Flavan-3-ol Derivatives from Palaeotropical *Artocarpus lacucha* Buch.-Ham Possess Radical Scavenging and Antiproliferative Properties. Molecules.

[13] Rahman A., Bishayee K., Sadra A., Huh S.-O. (2017). Oxyresveratrol activates parallel apoptotic and autophagic cell death pathways in neuroblastoma cells. Biochim. Biophys. Acta. Gen. Subj..

[14] Radapong S., Chan K., Sarker S.D., Ritchie K.J. (2021). Oxyresveratrol Modulates Genes Associated with Apoptosis, Cell Cycle Control and DNA Repair in MCF-7 Cells. Front. Pharmacol..

[15] Yang Y., Zhang G., Li C., Wang S., Shu X. (2019). Metabolic profile and structure-activity relationship of resveratrol and its analogs in human bladder cancer cells. Cancer Manag. Res..

[16] Sunilkumar D., Drishya G., Chandrasekharan A., Shaji S.K., Bose C., Jossart J., Perry J.J.P., Mishra N., Kumar G.B., Nair B.G. (2020). Oxyresveratrol drives caspase-independent apoptosis-like cell death in MDA-MB-231 breast cancer cells through the induction of ROS. Biochem. Pharmacol..

[17] Lv T., Jian Z., Li D., Ao R., Yu B. (2020). Oxyresveratrol induces apoptosis and inhibits cell viability via inhibition of the STAT3 signaling pathway in Saos2 cells. Mol. Med. Rep..

[18] Li R., Song Y., Ji Z., Li L., Zhou L. (2020). Pharmacological biotargets and the molecular mechanisms of oxyresveratrol treating colorectal cancer: Network and experimental analyses. BioFactors.

[19] Lin T.A., Lin W.S., Chou Y.C., Nagabhushanam K., Ho C.T., Pan M.H. (2021). Oxyresveratrol inhibits human colon cancer cell migration through regulating epithelial-mesenchymal transition and microRNA. Food Funct..

[20] Liu Y., Ren W., Bai Y., Wan L., Sun X., Liu Y., Xiong W., Zhang Y., Zhou L. (2018). Oxyresveratrol prevents murine H22 hepatocellular carcinoma growth and lymph node metastasis via inhibiting tumor angiogenesis and lymphangiogenesis. J. Nat. Med..

[21] Mutiah R., Sari R., Firsyaradha W., Listiyana A., Rahmawati A. (2020). Activity and Toxicity of *Eleutherine palmifolia* (L.) Merr. Extract on BALB/c Mice Colitis-Associated Colon Cancer Model. Asian Pac. J. Cancer Prev..

[22] Matencio A., Dhakar N., Bessone F., Musso G., Cavalli R., Dianzani C., García-Carmona F., López-Nicolás J.M., Trotta F. (2020). Study of oxyresveratrol complexes with insoluble cyclodextrin based nanosponges: Developing a novel way to obtain their complexation constants and application in an anticancer study. Carbohydr. Polym..

[23] Kim Y.M., Yun J., Lee C.K., Lee H., Min K.R., Kim Y. (2002). Oxyresveratrol and hydroxystilbene compounds: Inhibitory effect on tyrosinase and mechanism of action. J. Biol. Chem..

[24] Nimmanpisut S., Chudapongse P., Ratanabanangkoon K. (1976). Effects of 2, 4, 3′, 5′-tetrahydr oxystilbene on oxidative phosphorylation by rat liver mitochondria. Biochem. Pharmacol..

[25] Chatsumpun M., Chuanasa T., Sritularak B., Likhitwitayawuid K. (2011). Oxyresveratrol protects against DNA damage induced by photosensitized ribo-flavin. Nat. Prod. Commun..

[26] Wang Y.C., Chun W.U., Chen H., Zheng Y., Huang X.Z. (2011). Antioxidant activities of resveratrol, oxyresveratrol, esveratrol, mulberroside a from cortex mori. Food Sci..

[27] Starkov A.A. (2008). The role of mitochondria in reactive oxygen species metabolism and signaling. Ann. N.Y. Acad. Sci..

[28] Yokoo K., Yamamoto Y., Suzuki T. (2021). Ammonia impairs tight junction barriers by inducing mitochondrial dysfunction in Caco-2 cells. FASEB J..

[29] Ali R., Islamuddin M., Tabrez S., Alaidarous M.A., Alshehri B.M., Banawas S., Bin Dukhyil A.A., Rub A. (2021). Embilica officinalis L. inhibits the growth and proliferation of Leishmania donovani through the induction of ultrastructural changes, mitochondrial dysfunction, oxidative stress and apoptosis-like cell death. Biomed. Pharmacother..

[30] Pereira R.A., Pires A.D.R.A., Echevarria A., Sousa-Pereira D., Noleto G.R., Cadena S.M.S.C. (2021). The toxicity of 1,3,4-thiadiazolium mesoionic derivatives on hepatocarcinoma cells (HepG2) is associated with mitochondrial dysfunction. Chem. Biol. Interact..

[31] O’Brien P.J., Siraki A.G., Shangari N. (2005). Aldehyde sources, metabolism, molecular toxicity mechanisms, and possible effects on human health. Crit. Rev. Toxicol..

[32] Burcham P.C. (1998). Genotoxic lipid peroxidation products: Their DNA damaging properties and role in formation of endogenous DNA adducts. Mutagenesis.

[33] Tann A.W., Boldogh I., Meiss G., Qian W., Szczesny B. (2011). Apoptosis induced by persistent single-strand breaks in mitochondrial genome: Critical role of EXOG (5’-EXO/end onuclease) in their repair. J. Biol. Chem..

[34] Szczesny B., Módis K., Yanagi K., Coletta C., Le Trionnaire S., Perry A., Wood M.E., Whiteman M., Szabo C. (2014). AP39, a novel mitochondria-targeted hydrogen sulfide donor, stimulates cellular bioenergetics, exerts cytoprotective effects and protects against the loss of mitochondrial DNA integrity in oxidatively stressed endothelial cells in vitro. Nitric Oxide.

[35] Kawanishi M., Matsuda T., Sasaki G., Hideki Takebe H. (1998). A spectrum of mutations induced by crotonaldehyde in shuttle vector plasmids propagated in human cells. Carcinogenesis.

[36] Su Y., Sun C., Chen Y., Liu Y., Jing S., Li N., Xin S. (2019). Toxic trans-crotonaldehyde in mitochondria intercepted by oxyresveratrol contributing to anticancer. IUBMB Life.

[37] Passos C.L.A., Ferreira C., de Carvalho A.G.A., Silva J.L., Garrett R., Fialho E. (2024). Oxyresveratrol in Breast Cancer Cells: Synergistic Effect with Chemotherapeutics Doxorubicin or Melphalan on Proliferation, Cell Cycle Arrest, and Cell Death. Pharmaceutics.

[38] Ji K., Cho Y.S., Kim Y.T. (2017). Tyrosinase Inhibitory and Anti-oxidative Effects of Lactic Acid Bacteria Isolated from Dairy Cow Feces. Probiotics Antimicrob. Protein.

[39] Yanagihara M., Yoshimatsu M., Inoue A., Kanno T., Tatefuji T., Hashimoto K. (2012). Inhibitory Effect of Gnetin C, a Resveratrol Dimer from Melinjo (Gnetum gnemon), on Tyrosinase Activity and Melanin Biosynthesis. Biol. Pharm. Bull..

[40] Zhang L., Tao G., Chen J., Zheng Z.P. (2016). Characterization of a New Flavone and Tyrosinase Inhibition Constituents from the Twigs of *Morus alba* L.. Molecules.

[41] Panichakul T., Rodboon T., Suwannalert P., Tripetch C., Rungruang R., Boohuad N., Youdee P. (2020). Additive Effect of a Combination of Artocarpus lakoocha and Glycyrrhiza glabra Extracts on Tyrosinase Inhibition in Melanoma B16 Cells. Pharmaceuticals.

[42] Chaita E., Lambrinidis G., Cheimonidi C., Agalou A., Beis D., Trougakos I., Mikros E., Skaltsounis A.L., Aligiannis N. (2017). Anti-Melanogenic Properties of Greek Plants. A Novel Depigmenting Agent from Morus alba Wood. Molecules.

[43] Li J., Lin Z., Tang X., Liu G., Chen Y., Zhai X., Huang Q., Cao Y. (2020). Oxyresveratrol extracted from Artocarpus heterophyllus Lam. inhibits tyrosinase and age pigments in vitro and in vivo. Food Funct..

[44] Promden W., Chanvorachote P., Viriyabancha W., Sintupachee S., De-Eknamkul W. (2024). *Maclura cochinchinensis* (Lour.) Corner Heartwood Extracts Containing Resveratrol and Oxyresveratrol Inhibit Melanogenesis in B16F10 Melanoma Cells. Molecules.

[45] Çakır M., Saçmacı H. (2024). The relationship of salusins with Parkinson's Disease, Alzheimer's Disease, and acute ischemic stroke: A preliminary study. Neurosci. Lett..

[46] Huang P., Zhang L.-Y., Tan Y.-Y., Chen S.-D. (2023). Links between COVID-19 and Parkinson's disease/Alzheimer's disease: Reciprocal impacts, medical care strategies and underlying mechanisms. Transl. Neurodegener..

[47] Sprenkle N.T., Sims S.G., Cristina L.S., Meares G.P. (2017). Endoplasmic reticulum stress and inflammation in the central nervous system. Mol. Neurodegener..

[48] Zhang X., Bian J.S. (2014). Hydrogen sulfide: A neuromodulator and neuroprotectant in the central nervous system. ACS Chem. Neurosci..

[49] Zhong H., Yu H., Chen J., Sun J., Zhong Y. (2020). Hydrogen Sulfide and Endoplasmic Reticulum Stress: A Potential Therapeutic Target for Central Nervous System Degeneration Diseases. Front. Pharmacol..

[50] Shah A., Chao J., Legido-Quigley C., Chang R.C.-C. (2019). Oxyresveratrol exerts ATF4- and Grp78-mediated neuroprotection against endoplasmic reticulum stress in experimental Parkinson's disease. Nutr. Neurosci..

[51] Shah A., Ho Y., Ng K., Wang M., Legido-Quigley C., Chang R.C. (2020). Neuroprotective effects of oxyresveratrol on 6-hydroxydopamine on medial forebrain bundles in a rat model of Parkinson disease: Abridged secondary publication. Hong Kong Med. J..

[52] Rahman M., Cho Y., Nam G., Rhim H. (2021). Antioxidant Compound, Oxyresveratrol, Inhibits APP Production through the AMPK/ULK1/mTOR-Mediated Autophagy Pathway in Mouse Cortical Astrocytes. Antioxidants.

[53] Sangsen Y., Sooksawate T., Likhitwitayawuid K., Sritularak B., Wiwattanapatapee R. (2018). A Self-Microemulsifying Formulation of Oxyresveratrol Prevents Amyloid Beta Protein-Induced Neurodegeneration in Mice. Planta Medica.

[54] Wang X., Zhu X., Li X., Li Z., Mao Y., Zhang S., Liu X., Liu X., Liu Y., Cao F. (2023). Transcriptomic and metabolomic analyses provide insights into the attenuation of neuroinflammation by nervonic acid in MPTP-stimulated PD model mice. Food Funct..

[55] Lemprière S. (2023). Neuroinflammation, not amyloid-β deposition, associated with brain network dysfunction in AD. Nat. Rev. Neurol..

[56] Hankittichai P., Lou H., Wikan N., Smith D.R., Potikanond S., Nimlamool W. (2020). Oxyresveratrol Inhibits IL-1β-Induced Inflammation via Suppressing AKT and ERK1/2 Activation in Human Microglia, HMC3. Int. J. Mol. Sci..

[57] Wang L., Zhao H., Wang L., Tao Y., Du G., Guan W., Liu J., Brennan C., Ho C.T., Li S. (2020). Effects of Selected Resveratrol Analogues on Activation and Polarization of Lipopolysaccharide-Stimulated BV-2 Microglial Cells. J. Agric. Food Chem..

[58] Cho H.M., Ha T.K.Q., Pham H.T.T., An J.-P., Huh J., Lee B.-W., Lee H.J., Oh W.K. (2019). Oligostilbenes from the leaves of Gnetum latifolium and their biological potential to inhibit neuroinflammation. Phytochemistry.

[59] Lee H.J., Feng J.H., Sim S.M., Lim S.S., Lee J.Y., Suh H.W. (2019). Effects of resveratrol and oxyresveratrol on hippocampal cell death induced by kainic acid. Anim. Cells Syst..

[60] Wu Y., Li S., Liu J., Liu X., Ruan W., Lu J., Liu Y., Lawson T., Shimoni O., Lovejoy D.B. (2018). Stilbenes from *Veratrum maackii* Regel Protect against Ethanol-Induced DNA Damage in Mouse Cerebellum and Cerebral Cortex. ACS Chem. Neurosci..

[61] Henderson K., Lewis K.H., Sloan C., Bessesen D.H., Arterburn D. (2024). Effectiveness and safety of drugs for obesity. BMJ.

[62] Cohen P., Kajimura S. (2021). The cellular and functional complexity of thermogenic fat. Nat. Rev. Mol. Cell Biol..

[63] Jain R., Simcox J. (2022). Igniting adipocyte thermogenesis. Cell.

[64] Li Y., Wang D., Ping X., Zhang Y., Zhang T., Wang L., Jin L., Zhao W., Guo M. (2022). Local hyperthermia therapy induces browning of white fat and treats obesity. Cell.

[65] Pan M.H., Koh Y.C., Lee T.L., Wang B., Ho C. (2019). Resveratrol and Oxyresveratrol Activate Thermogenesis via Different Transcriptional Coactivators in High-Fat Diet-Induced Obese Mice. J. Agric. Food Chem..

[66] Tan H., Tse I., Li E., Wang M. (2017). Oxyresveratrol Supplementation to C57bl/6 Mice Fed with a High-Fat Diet Ameliorates Obesity-Associated Symptoms. Nutrients.

[67] Lee D.H., Seo M.J., Kim S., Chang S.H., Yang D.K., Hwang Y.J., Hwang K.A., Ha T.S., Yun U.J., Park K.W. (2018). Oxyresveratrol Increases Energy Expenditure through Foxo3a-Mediated Ucp1 Induction in High-Fat-Diet-Induced Obese Mice. Int. J. Mol. Sci..

[68] Cione E., Abrego Guandique D.M., Caroleo M.C., Luciani F., Colosimo M., Cannataro R. (2023). Liver Damage and microRNAs: An Update. Curr. Mol. Biol..

[69] Brandon-Warner E., Feilen N.A., Culberson C.R., Field C.O., de Lemos A.S., Russo M.W., Schrum L.W. (2016). Processing of miR17-92 Cluster in Hepatic Stellate Cells Promotes Hepatic Fibrogenesis During Alcohol-Induced Injury. Alcohol. Clin. Exp. Res..

[70] Choi H.Y., Lee J.-H., Jegal K.H., Cho I.J., Kim Y.W., Kim S.C. (2016). Oxyresveratrol abrogates oxidative stress by activating ERK-Nrf2 pathway in the liver. Chem. Biol. Interact..

[71] Jia Y., Peng Y., Zhao Y., Cheng X.F., Zhou Y., Chai C.L., Zeng L.S., Pan M.H., Xu L. (2019). Comparison of the Hepatoprotective Effects of the Three Main Stilbenes from Mulberry Twigs. J. Agric. Food Chem..

[72] Jia Y.-N., Lu H.-P., Peng Y.-L., Zhang B.-S., Gong X.-B., Su J., Zhou Y., Pan M.-H., Xu L. (2018). Oxyresveratrol prevents lipopolysaccharide/d-galactosamine-induced acute liver injury in mice. Int. Immunopharmacol..

[73] Federico S., Maria P.R., Miriam L., Francesco M., Maria A.A., Alfio D., Elia C., Ester C., Nunzio I., Loredana L. (2024). SIRT5 rs12216101 T>G variant is associated with liver damage and mitochondrial dysfunction in patients with non-alcoholic fatty liver disease. J. Hepatol..

[74] Lee J.H., Baek S.Y., Jang E.J., Ku S.K., Kim K.M., Ki S.H., Kim C.E., Park K.I., Kim S.C., Kim Y.W. (2018). Oxyresveratrol ameliorates nonalcoholic fatty liver disease by regulating hepatic lipogenesis and fatty acid oxidation through liver kinase B1 and AMP-activated protein kinase. Chem. Biol. Interact..

[75] Lee E.H., Park K.I., Kim K.Y., Lee J.H., Jang E.J., Ku S.K., Kim S.C., Suk H.Y., Park J.Y., Baek S.Y. (2019). Liquiritigenin inhibits hepatic fibrogenesis and TGF-β1/Smad with Hippo/YAP signal. Phytomedicine.

[76] Ye D., Guo S., Al-Sadi R., Ma T.Y. (2011). MicroRNA regulation of intestinal epithelial tight junction permeability. Gastroenterology.

[77] Ganapathy A.S., Saha K., Wang A., Arumugam P., Dharmaprakash V., Yochum G., Koltun W., Nighot M., Perdew G., Thompson T.A. (2023). Alpha-tocopherylquinone differentially modulates claudins to enhance intestinal epithelial tight junction barrier via AhR and Nrf2 pathways. Cell Rep..

[78] Abdulqadir R., Engers J., Al-Sadi R. (2023). Role of Bifidobacterium in Modulating the Intestinal Epithelial Tight Junction Barrier: Current Knowledge and Perspectives. Curr. Dev. Nutr..

[79] Hwang D., Jo H., Hwang S., Kim J.K., Kim I.H., Lim Y.H. (2017). Conditioned medium from LS 174T goblet cells treated with oxyresveratrol strengthens tight junctions in Caco-2 cells. Biomed. Pharmacother..

[80] Jo H., Hwang D., Kim J.-K., Lim Y.-H. (2017). Oxyresveratrol improves tight junction integrity through the PKC and MAPK signaling pathways in Caco-2 cells. Food Chem. Toxicol..

[81] Yeom J., Ma S., Kim J.-K., Lim Y.H. (2021). Oxyresveratrol Ameliorates Dextran Sulfate Sodium-Induced Colitis in Rats by Suppressing Inflammation. Molecules.

[82] Hwang D., Jo H., Ma S., Lim Y.H. (2018). Oxyresveratrol stimulates mucin production in an NAD-dependent manner in human intestinal goblet cells. Food Chem. Toxicol..

[83] Yeom J., Ma S., Lim Y.-H. (2020). Oxyresveratrol Induces Autophagy via the ER Stress Signaling Pathway, and Oxyresveratrol-Induced Autophagy Stimulates MUC2 Synthesis in Human Goblet Cells. Antioxidants.

[84] Zakova T., Rondevaldova J., Bernardos A., Landa P., Kokoska L. (2018). The relationship between structure and in vitro antistaphylococcal effect of plant-derived stilbenes. Acta Microbiol. Immunol. Hung..

[85] Wu J., Fan Y., Wang X., Jiang X., Zou J. (2020). Effects of the natural compound, oxyresveratrol, on the growth of Streptococcus mutans, and on biofilm formation, acid production, and virulence gene expression. Eur. J. Oral. Sci..

[86] Wu J., Jiang X., Yang Q., Zhang Y., Wang C., Huang R. (2022). Inhibition of Streptococcus mutans Biofilm Formation by the Joint Action of Oxyresveratrol and *Lactobacillus casei*. Appl. Environ. Microbiol..

[87] Wu J., Yang Q., Jiang X., Fan Y., Zhang Y., Huang R. (2020). Oxyresveratrol promotes biofilm formation, cell attachment and aggregation of *Streptococcus gordonii* in the presence of sucrose. FEMS Microbiol. Lett..

[88] Joung D., Mun S., Choi S., Kang O.H., Kim S.B., Lee Y.S., Zhou T., Kong R., Choi J.G., Shin D.W. (2016). Antibacterial activity of oxyresveratrol against methicillin-resistant *Staphylococcus aureus* and its mechanism. Exp. Ther. Med..

[89] Joung D., Choi S., Kang O., Kim S.B., Mun S.H., Seo Y.S., Kang D.H., Gong R., Shin D.W., Kim Y.C. (2015). Synergistic effects of oxyresveratrol in conjunction with antibiotics against methicillin-resistant *Staphylococcus aureus*. Mol. Med. Rep..

[90] Lee J.H., Kim Y.G., Raorane C.J., Ryu S.Y., Shim J.J., Lee J. (2019). The anti-biofilm and anti-virulence activities of trans- resveratrol and oxyresveratrol against uropathogenic *Escherichia coli*. Biofouling.

[91] Kim S., Lee D. (2018). DNA oxyresveratrol-induced DNA cleavage triggers apoptotic response in Candida albicans. Microbiology.

[92] Lu H.P., Jia Y.N., Peng Y.L., Yu Y., Sun S.L., Yue M.T., Pan M.H., Zeng L.S., Xu L. (2017). Oxyresveratrol, a Stilbene Compound from *Morus alba* L. Twig Extract Active Against *Trichophyton rubrum*. Phytother. Res..

[93] Lin W.W., Karin M. (2007). A cytokine-mediated link between innate immunity, inflammation, and cancer. J. Clin. Investig..

[94] Wang X., Zhang J., Yang L., Wang T., Duan G., Gu Z., Li Y. (2024). Eumelanin-like Poly(levodopa) Nanoscavengers for Inflammation Disease Therapy. Biomacromolecules.

[95] Olive L. (2014). Inflammation: Regulating ROS. Nat. Rev. Immunol..

[96] Wei J., Chen J., Pais E., Wang T.Y., Miao L., Li L., Li L.Y., Qiu F., Hu L.M., Gao X.M. (2017). Oxyresveratrol is a Phytoestrogen Exerting Anti-inflammatory Effects Through NF-κB and Estrogen Receptor Signaling. Inflammation.

[97] Thaweesest W., Buranasudja V., Phumsuay R., Muangnoi C., Vajragupta O., Sritularak B., Rashatasakhon P., Rojsitthisak P. (2022). Anti-Inflammatory Activity of Oxyresveratrol Tetraacetate, an Ester Prodrug of Oxyresveratrol, on Lipopolysaccharide-Stimulated RAW264.7 Macrophage Cells. Molecules.

[98] Tran H.G., Shuayprom A., Kueanjinda P., Leelahavanichkul A., Wongsinkongman P., Chaisomboonpan S., Tawatsin A., Ruchusatsawat K., Wongpiyabovorn J. (2023). Oxyresveratrol Attenuates Inflammation in Human Keratinocyte via Regulating NF-kB Signaling and Ameliorates Eczematous Lesion in DNCB-Induced Dermatitis Mice. Pharmaceutics.

[99] Hornedo-Ortega R., Jourdes M., Da Costa G., Courtois A., Gabaston J., Teissedre P.L., Richard T., Krisa S. (2022). In Vitro Oxyresveratrol and Gnetol Glucuronide Metabolites: Chemical Production, Structural Identification, Metabolism by Human and Rat Liver Fractions, and Anti-inflammatory Properties. J. Agric. Food Chem..

[100] Wongwat T., Srihaphon K., Pitaksutheepong C., Boonyo W., Pitaksuteepong T. (2019). Suppression of inflammatory mediators and matrix metalloproteinase (MMP)-13 by *Morus alba* stem extract and oxyresveratrol in RAW 264.7 cells and C28/I2 human chondrocytes. J. Tradit. Complement. Med..

[101] Jongkon N., Seaho B., Tayana N., Prateeptongkum S., Duangdee N., Jaiyong P. (2022). Computational Analysis and Biological Activities of Oxyresveratrol Analogues, the Putative Cyclooxygenase-2 Inhibitors. Molecules.

[102] Aziz R.S., Siddiqua A., Shahzad M., Shabbir A., Naseem N. (2019). Oxyresveratrol ameliorates ethanol-induced gastric ulcer via downregulation of IL-6, TNF-α, NF-ĸB, and COX-2 levels, and upregulation of TFF-2 levels. Biomed. Pharmacother..

[103] Wikan N., Hankittichai P., Thaklaewphan P., Potikanond S., Nimlamool W. (2021). Oxyresveratrol Inhibits TNF-α-Stimulated Cell Proliferation in Human Immortalized Keratinocytes (HaCaT) by Suppressing AKT Activation. Pharmaceutics.

[104] Davidoff F. (1997). Blood sugar, disease, and nondisease. Ann. Intern. Med..

[105] Xiao Y., Hu Y., Du J. (2019). Controlling blood sugar levels with a glycopolymersome. Mater. Horiz..

[106] Bondy S.C., Wu M., Prasad K.N. (2020). Alternatives to Insulin for the Regulation of Blood Sugar Levels in Type 2 Diabetes. Int. J. Mol. Sci..

[107] Park S., Jin B., Shin J., Adisakwattana S., Kwon O. (2017). Standardized Mori ramulus extract improves insulin secretion and insulin sensitivity in C57BLKS/J db/db mice and INS-1 cells. Biomed. Pharmacother..

[108] Zheng Y., He H., Wei X., Ge S., Lu Y.H. (2016). Comparison of Regulation Mechanisms of Five Mulberry Ingredients on Insulin Secretion under Oxidative Stress. J. Agric. Food Chem..

[109] Wang W., Yang R., Yao H., Wu Y., Jia A.Q. (2019). Inhibiting the formation of advanced glycation end-products by three stilbenes and the identification of their adducts. Food Chem..

[110] He H., Yu W., Yang J., Ge S., Lu Y. (2016). Multiple Comparisons of Glucokinase Activation Mechanisms of Five Mulberry Bioactive Ingredients in Hepatocyte. J. Agric. Food Chem..

[111] Ahn E., Lee J., Jeon Y.-H., Choi S.-W., Kim E. (2017). Anti-diabetic effects of mulberry *(Morus alba* L.) branches and oxyresveratrol in streptozotocin-induced diabetic mice. Food Sci. Biotechnol..

[112] Wongon M., Limpeanchob N. (2020). Artocarpus lakoocha Inhibitory effect of Roxb and oxyresveratrol on α-glucosidase and sugar digestion in Caco-2 cells. Heliyon.

[113] Wongon M., Limpeanchob N. (2021). Artocarpus lacucha Extract and Oxyresveratrol Inhibit Glucose Transporters in Human Intestinal Caco-2 Cells. Planta Medica.

[114] Jarosova V., Vesely O., Marsik P., Jaimes J.D., Smejkal K., Kloucek P., Havlik J. (2019). Metabolism of Stilbenoids by Human Faecal Microbiota. Molecules.

[115] Prakash V., Krishnan A.S., Ramesh R., Bose C., Pillai G.G., Nair B.G., Pal S. (2021). Synergistic Effects of Limosilactobacillus fermentum ASBT-2 with Oxyresveratrol Isolated from Coconut Shell Waste. Foods.

[116] Hu X., Liang Y., Zhao B., Wang Y. (2019). Oxyresveratrol protects human lens epithelial cells against hydrogen peroxide-induced oxidative stress and apoptosis by activation of Akt/HO-1 pathway. J. Pharmacol. Sci..

[117] Yan M., Man S., Sun B., Ma L., Guo L., Huang L., Gao W. (2023). Gut liver brain axis in diseases: The implications for therapeutic interventions. Signal Transduct. Target. Ther..

[118] Sepehrinezhad A., Shahbazi A., Joghataei M.T., Larsen F.S., Negah S.S. (2023). Inhibition of autotaxin alleviates pathological features of hepatic encephalopathy at the level of gut-live-brain axis: An experimental and bioinformatic study. Cell Death Dis..

[119] Simpson C.A., Diaz-Arteche C., Eliby D., Schwartz O.S., Simmons J.G., Cowan C.S. (2021). The gut microbiota in anxiety and depression—A systematic review. Clin. Psychol. Rev..

[120] Honarpisheh P., Bryan R.M., McCullough L.D. (2022). Aging microbiota-gut-brain axis in stroke risk and outcome. Circ. Res..

[121] Doifode T., Giridharan V.V., Generoso J.S., Bhatti G., Collodel A., Schulz P.E., Forlenza O.V., Barichello (2021). The impact of the microbiota-gut-brain axis on Alzheimer’s disease pathophysiology. Pharmacol. Res..

[122] Tan A.H., Lim S.Y., Lang A.E. (2022). The microbiome-gut-brain axis in Parkinson disease—From basic research to the clinic. Nat. Rev. Neurol..

[123] Trovato F.M., Zia R., Artru F., Mujib S., Jerome E., Cavazza A., Coen M., Wilson I., Holmes E., Morgan P. (2022). Lysophosphatidylcholines modulate immunoregulatory checkpoints in peripheral monocytes and are associated with mortality in people with acute liver failure. J. Hepatol..

[124] Nie C., Zhang L., Chen X., Li Y., Ha F., Liu H., Han T. (2020). Autotaxin: An early warning biomarker for acute-on-chronic liver failure. J. Clin. Transl. Hepatol..

[125] Fujimori N., Umemura T., Kimura T., Tanaka N., Sugiura A., Yamazaki T., Joshita S., Komatsu M., Usami Y., Sano K. (2018). Serum autotaxin levels are correlated with hepatic fibrosis and ballooning in patients with non-alcoholic fatty liver disease. World J. Gastroenterol..

[126] Fujino H., Tanaka M., Imamura M., Morio K., Ono A., Nakahara T., Murakami E., Kawaoka T., Takahashi S., Miki D. (2019). Pruritus in patients with chronic liver disease and serum autotaxin levels in patients with primary biliary cholangitis. BMC Gastroenterol..

[127] Joshi L., Plastira I., Bernhart E., Reicher H., Triebl A., Köfeler H.C., Sattler W. (2021). Inhibition of Autotaxin and Lysophosphatidic Acid Receptor 5 Attenuates Neuroinflammation in LPS-Activated BV-2 Microglia and a Mouse Endotoxemia Model. Int. J. Mol. Sci..

[128] Plastira I., Bernhart E., Joshi L., Koyani C.N., Strohmaier H., Reicher H., Malle E., Sattler W. (2020). MAPK signaling determines lysophosphatidic acid (LPA)-induced inflammation in microglia. J. Neuroinflammation.

[129] Roy S., Chakrabarti M., Dasgupta H., Mahale A., Tripathi S., Sharma V., Banerjee M., Kulkarni O.P. (2022). Inhibition of autotaxin ameliorates LPA-mediated neuroinflammation and alleviates neurological dysfunction in acute hepatic encephalopathy. ACS Chem. Neurosci..

[130] Bajaj J.S. (2019). Alcohol, liver disease and the gut microbiota. Nat. Rev. Gastroenterol. Hepatol..

[131] Kang Y., Cai Y., Yang Y. (2022). The gut microbiome and hepatocellular carcinoma: Implications for early diagnostic biomarkers and novel therapies. Liver Cancer.

[132] Bence K.K., Birnbaum M.J. (2021). Metabolic drivers of non-alcoholic fatty liver disease. Mol. Metab..

[133] Sun X., Lv Y., Huang L., Gao H., Ren C., Li J., Bie M., Li W., Koike K., So K.-F. (2020). Pro-inflammatory cytokines serve as communicating molecules between the liver and brain for hepatic encephalopathy pathogenesis and Lycium barbarum polysaccharides protection. J. Ethnopharmacol..

[134] Sepehrinezhad A., Zarifkar A., Namvar G., Shahbazi A., Williams R. (2020). Astrocyte swelling in hepatic encephalopathy: Molecular perspective of cytotoxic edema. Metab. Brain Dis..

[135] Trapecar M., Wogram E., Svoboda D., Communal C., Omer A., Lungjangwa T. (2021). Human physiomimetic model integrating microphysiological systems of the gut, liver, and brain for studies of neurodegenerative diseases. Sci. Adv..

[136] Baumler A.J., Sperandio V. (2016). Interactions between the microbiota and pathogenic bacteria in the gut. Nature.

[137] Snigdha S., Ha K., Tsai P., Dinan T.G., Bartos J.D., Shahid M. (2022). Probiotics: Potential novel therapeutics for microbiota-gut-brain axis dysfunction across gender and lifespan. Pharm. Ther..

[138] Cao Y.-Y., Wang Z., Wang Z.-H., Jiang X.-G., Lu W.-H. (2021). Inhibition of miR-155 alleviates sepsis-induced inflammation and intestinal barrier dysfunction by inactivating NF-kappaB signaling. Int. Immunopharmacol..

[139] Browne I.M., André F., Chandarlapaty S., Carey L., Turner N.C. (2024). Optimal targeting of PI3K-AKT and mTOR in advanced oestrogen receptor-positive breast cancer. Lancet Oncol..

[140] Hoxhaj G., Manning B.D. (2020). The PI3K-AKT network at the interface of oncogenic signalling and cancer metabolism. Nat. Rev. Cancer.

[141] Jimi E., Aoki K., Saito H., D'Acquisto F., May M.J., Nakamura I., Sudo T., Kojima T., Okamoto F., Fukushima H. (2004). Selective inhibition of NF-kappa B blocks osteoclastogenesis and prevents inflammatory bone destruction in vivo. Nat. Med..

[142] Wang T., Fu X., Chen Q., Patra J.K., Wang D., Wang Z., Gai Z. (2019). Arachidonic Acid Metabolism and Kidney Inflammation. Int. J. Mol. Sci..

[143] Yuan J., Yan F., Li W., Yuan G. (2022). Network pharmacological analysis of Xuefu Zhuyu decoction in the treatment of atherosclerosis. Front. Pharmacol..

[144] Zhao F., Qin J., Liang Y., Zhou R. (2021). Exploring anti-liver cancer targets and mechanisms of oxyresveratrol: In silico and verified findings. Bioengineered.

[145] Tan B., Wikan N., Lin S., Thaklaewphan P., Potikanond S., Nimlamool W. (2024). Inhibitory actions of oxyresveratrol on the PI3K/AKT signaling cascade in cervical cancer cells. Biomed. Pharmacother..

[146] Casagrande B., Pisani L., Estadella D. (2021). AMPK in the gut-liver-brain axis and its influence on OP rats in an HSHF intake and WTD rat model. Pflug. Arch. Eur. J. Physiol..

[147] Ansari A., Bose S., Lim S., Wang J., Choi Y., Kim H. (2020). Scutellaria baicalensis Combination of and Metformin Ameliorates Diet-Induced Metabolic Dysregulation in Mice via the Gut-Liver-Brain Axis. Am. J. Chin. Med..

[148] Brescia P., Rescigno M. (2021). The gut vascular barrier: A new player in the gut-liver-brain axis. Trends Mol. Med..

[149] Hu S., Luo L., Zeng L. (2023). Tea combats circadian rhythm disorder syndrome via the gut-liver-brain axis: Potential mechanisms speculated. Crit. Rev. Food Sci. Nutr..

[150] Giuffrè M., Moretti R. (2023). The Gut-Liver-Brain Axis: From the Head to the Feet. Int. J. Mol. Sci..

[151] Tache Y., Saavedra J.M. (2022). Introduction to the Special Issue “The Brain-Gut Axis”. Cell. Mol. Neurobiol..

[152] Dogra N., Mani R.J., Katare D.P. (2021). The Gut-Brain Axis: Two Ways Signaling in Parkinson's Disease. Cell. Mol. Neurobiol..

[153] Li Y., Pan L., Zeng X., Zhang R., Li X., Li J., Xing H., Bao J. (2021). Ammonia exposure causes the imbalance of the gut-brain axis by altering gene networks associated with oxidative metabolism, inflammation and apoptosis. Ecotoxicol. Environ. Saf..

[154] Guenther C., Rothhammer V., Karow M., Winner B. (2021). The Gut-Brain Axis in Inflammatory Bowel Disease-Current and Future Perspectives. Int. J. Mol. Sci..

[155] Lee S.G., Lee D.G., Joo Y.H., Chung N. (2021). Synergistic inhibitory effects of the oxyresveratrol and dacarbazine combination against melanoma cells. Oncol. Lett..

[156] Mahamud N., Songvut P., Muangnoi C., Rodsiri R., Dahlan W., Tansawat R. (2023). Untargeted metabolomics reveal pathways associated with neuroprotective effect of oxyresveratrol in SH-SY5Y cells. Sci. Rep..

[157] Yin G., Pan C., Liu H., Dong C., Chang X., Zhou W., Wang S., Du Z. (2024). Oxyresveratrol Improves Cognitive Impairments and Episodic-like Memory through Modulating Neuroinflammation and PI3K-Akt Signaling Pathway in LPS-Induced Mice. Molecules.

[158] Alam N., Najnin H., Islam M., Shakya S., Khan I.M., Zaidi R. (2023). Biochemical and histopathological analysis after sub-chronic administration of oxyresveratrol in Wistar rats. Drug Chem. Toxicol..

[159] Dhakar N.K., Matencio A., Caldera F., Argenziano M., Cavalli R., Dianzani C., Zanetti M., López-Nicolás J.M., Trotta F. (2019). Comparative Evaluation of Solubility, Cytotoxicity and Photostability Studies of Resveratrol and Oxyresveratrol Loaded Nanosponges. Pharmaceutics.

[160] Lakshmi S., Raghu S.V., Elumalai P., Sivan S. (2021). Alkoxy glycerol enhanced activity of Oxyresveratrol in Alzheimer's disease by rescuing Tau protein. Neurosci. Lett..

[161] Sangsen Y., Wiwattanawongsa K., Likhitwitayawuid K., Sritularak B., Graidist P., Wiwattanapatapee R. (2016). Influence of surfactants in self-microemulsifying formulations on enhancing oral bioavailability of oxyresveratrol: Studies in Caco-2 cells and in vivo. Int. J. Pharm..

[162] Suzuki Y., Muangnoi C., Thaweesest W., Teerawonganan P., Na Bhuket P.R., Titapiwatanakun V., Yoshimura-Fujii M., Sritularak B., Likhitwitayawuid K., Rojsitthisak P. (2019). Exploring Novel Cocrystalline Forms of Oxyresveratrol to Enhance Aqueous Solubility and Permeability across a Cell Monolayer. Biol. Pharm. Bull..

[163] Liu T., Liu M., Liu H., Ren Y., Zhao Y., Yan H., Wang Q., Ning Z., Ding Z., Wang Z. (2021). Co-encapsulation of (-)-epigallocatechin-3-gallate and piceatannol/oxyresveratrol in β-lactoglobulin: Effect of ligand-protein binding on the antioxidant activity, stability, solubility and cytotoxicity. Food Funct..

